# An affordable and miniature ice coring drill for rapid acquisition of small iceberg samples

**DOI:** 10.1016/j.ohx.2020.e00101

**Published:** 2020-02-27

**Authors:** Steffen Thomsen, Mads Holm Hansen, Jeppe Pinholt Lillethorup, Frederik Sebastian Tirsgaard, Adam Flytkjær, Claus Melvad, Søren Rysgaard, Daniel F. Carlson

**Affiliations:** a School of Engineering, Aarhus University, Aarhus, Denmark; b Arctic Research Centre, Department of Bioscience, Aarhus University, Aarhus, Denmark; c Department of Earth, Ocean, and Atmospheric Science, Florida State University, Tallahassee, FL, USA; d Institute of Coastal Research, Helmholtz-Zentrum Geesthacht, Centre for Materials and Coastal Research, Geesthacht, Germany

**Keywords:** Iceberg, Sample, Core, Drill, Marine ecosystem, Greenland

## Abstract

Icebergs account for approximately half of the freshwater flux from the Greenland Ice Sheet and they can impact marine ecosystems by releasing nutrients and sediments into the ocean as they drift and melt. Parameterizing iceberg fluxes of nutrients and sediments to fjord and ocean waters remains a difficult task due to the complexity of ice-ocean interactions and is complicated by a lack of observations. Acquiring iceberg samples can be difficult and dangerous, as icebergs can break apart and roll without warning. Here we present open source design files for a small, lightweight ice coring drill that can be reproduced using modern computer numerical control (CNC) machining and 3D printing technology. This ice core drill can rapidly acquire small ice samples from icebergs and bergy bits using a standard commercial, off-the-shelf battery-operated hand drill. Design files and a recent field expedition to Northwest Greenland are described. Ice core collection required only 30 s, thereby minimizing risks to scientists.

## Graphical abstract

**Figure:**
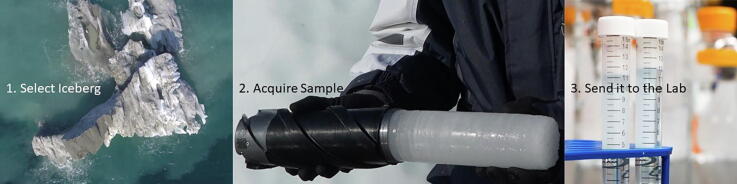

**Specifications table:**

**Table:** 

Hardware name	Miniature mechanical ice drill
Subject area	Environmental, Planetary and Agricultural Sciences
Hardware type	Field measurements and sensors
Open source license	CC BY 4.0
Cost of hardware	220 euro
Source file repository	https://doi.org/10.17632/gkg6wjp844.1

## 1 Hardware in context

### 1.1 Motivation

Mass loss from the Greenland Ice Sheet (GrIS) has increased by nearly a factor of six since the 1980s [1], contributing to sea level rise [2], [3] and altering density stratification in fjords [4], [5]. Glacial meltwater runoff can impact fjord marine ecosystems [6], [7], [8], [9] and icebergs comprise approximately half of the freshwater flux into the ocean [10]. Icebergs release nutrients and sediments as they melt along their drift trajectory [11], [12], [13], [14], [15], [16], [17], [18], [19], [20], [21], [22], [23], [24] and emerging observational evidence suggests that they may also impact ocean microbial communities [25], [26].

The variability in the biogeochemical composition of icebergs needs to be adequately characterized to develop ecosystem models for polar regions [24]. Thus, a sufficient number of samples from multiple fjord systems and across the spectrum of iceberg size, shape, and sediment classes is required [24]. Ice islands and large (L > 100 m) icebergs are relatively stable and, as a result, measurements have been biased towards large and flat icebergs that permit helicopter landings [27], [28]. Indeed, large icebergs were even proposed as aircraft carriers during World War II [29]. Operations on or near smaller icebergs and bergy bits, however, are difficult and dangerous, as they are unstable and may break apart and/or overturn without warning [30]. Therefore, new sampling techniques that permit the safe retrieval of samples from smaller icebergs and bergy bits are required.

### 1.2 Summary of ice sampling methods

#### 1.2.1 Icebergs

The International Ice Patrol (IIP) began monitoring iceberg populations in the North Atlantic following the sinking of the *Titanic* in 1912 [12], [31]. In addition to monitoring iceberg populations to enhance safety at sea, the *Titanic* incident spurred interest in iceberg properties to understand their thermodynamic and mechanical properties [32], [33].

In the past few decades, offshore oil exploration in the North Atlantic has renewed interest in iceberg properties from the standpoint of iceberg management [34]. Ice samples were harvested from icebergs using chainsaws by helicopter and small boats to quantify mechanical properties [35], [34], [36]. Helicopters increase costs significantly and using a chainsaw introduces additional safety risks to the sampling procedure.

More recently, the increased mass loss from the Greenland Ice Sheet has drawn attention to the impacts of icebergs on marine ecosystems. In these studies, small samples (<0.5 m) were obtained either using ice axes or by fishing small bits of floating ice out of the water [16], [24]. Retrieving small, floating bits of ice is relatively safe but the sample has been bathed in seawater, which alters the exterior of the ice. Thus, a device that could rapidly retrieve ice samples of consistent size and weight from a small boat would greatly enhance our understanding of the role of icebergs in marine ecosystems.

#### 1.2.2 Ice drilling

Ice drilling systems have been used for over a century on glaciers, ice sheets, and in sea ice [37]. The majority of ice drilling programs, however, have focused on collecting relatively long cores. In fact, [37] considers ‘small’ systems to be those capable of drilling to depths of up to 50 m. The so-called ‘Chipmunk Drill’ [37], [38], [39] can be operated by a battery-powered hand drill and has two barrels with lengths of 15 cm and 50 cm. The Chipmunk drill is not commercially available nor are the plans in the public domain. Similarly, other small (0.5–1 m) hand drills are described in [37] but the design files necessary for reproduction are lacking.

Commercial products in this size range are also limited, likely due to a lack of demand. The Kovacs Mark II coring system is 1 m long and costs approximately $5000 (USD) [40]. While the Kovacs Mark II is easy to operate in a vertical orientation on a relatively flat surface, like an ice cap [41], its length and weight can make coring difficult when used in a horizontal orientation from a moving boat.

Thus, the lack of either a suitable commercial product or an open source design necessitated the development of an ice drill to fulfill our research objectives. Given that a sample of 0.5–1 kg is sufficient for the chemical analyses that motivate this paper, we require a miniature ice drilling system. In addition to collecting samples of a relatively consistent size, a miniature ice drilling system is easier and, perhaps, safer to operate from a pitching, rolling, and heaving small boat.

Here we present a small and lightweight ice coring drill design that can rapidly acquire samples from icebergs and bergy bits using a standard commercial, off-the-shelf battery-operated hand drill. The hand drilling system was developed in parallel with a drone-mounted ice sampler [42], which will allow researchers to acquire samples from icebergs when weather, technical problems, and/or regulations prevent the use of the drone.

We describe the design files and a recent field expedition to Northwest Greenland, where the miniature ice coring drill presented here was used to retrieve 11 samples. Sample acquisition took approximately 30 s, permitting ‘in-and-out’ sampling of freely drifting icebergs using a small boat. This rapid sampling capability reduces risks to researchers and their support equipment and will increase the number of iceberg samples available to develop robust statistical parameterizations of iceberg nutrient and sediment loads.

## 2 Hardware description

• Simple to assemble and operate
• Fast sample acquisition
• Lightweight and small

**Fig. 1:**
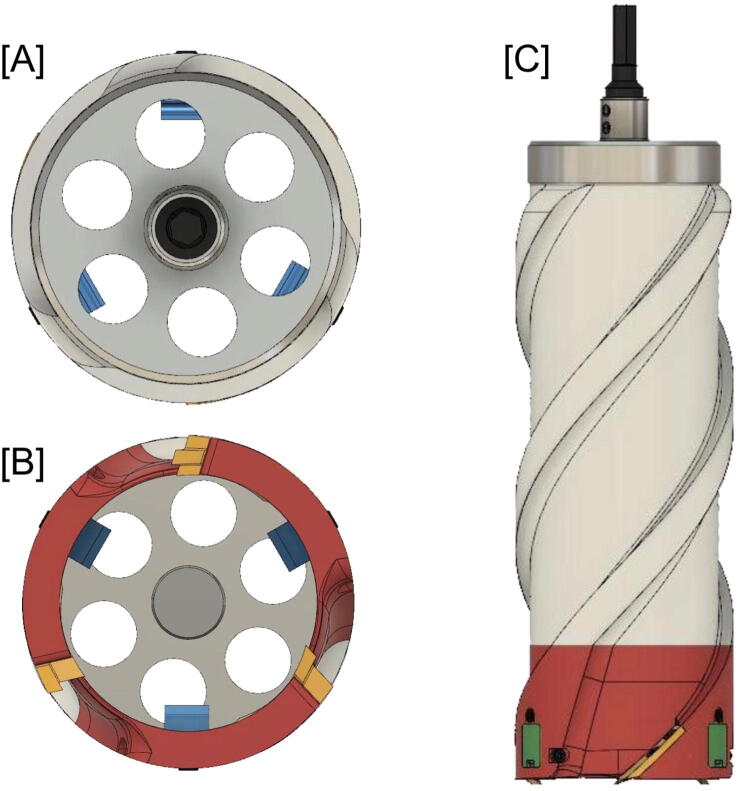
Color renderings that show top [A], bottom [B], and side view [C] of the ice coring drill design. The ice coring drill shaft, top, sleeve, and cutting head are colored black, silver, white, and red, respectively. On the cutting head, the core dogs, core dog covers, and cutting teeth are colored blue, green, and yellow, respectively.

**Fig. 2:**
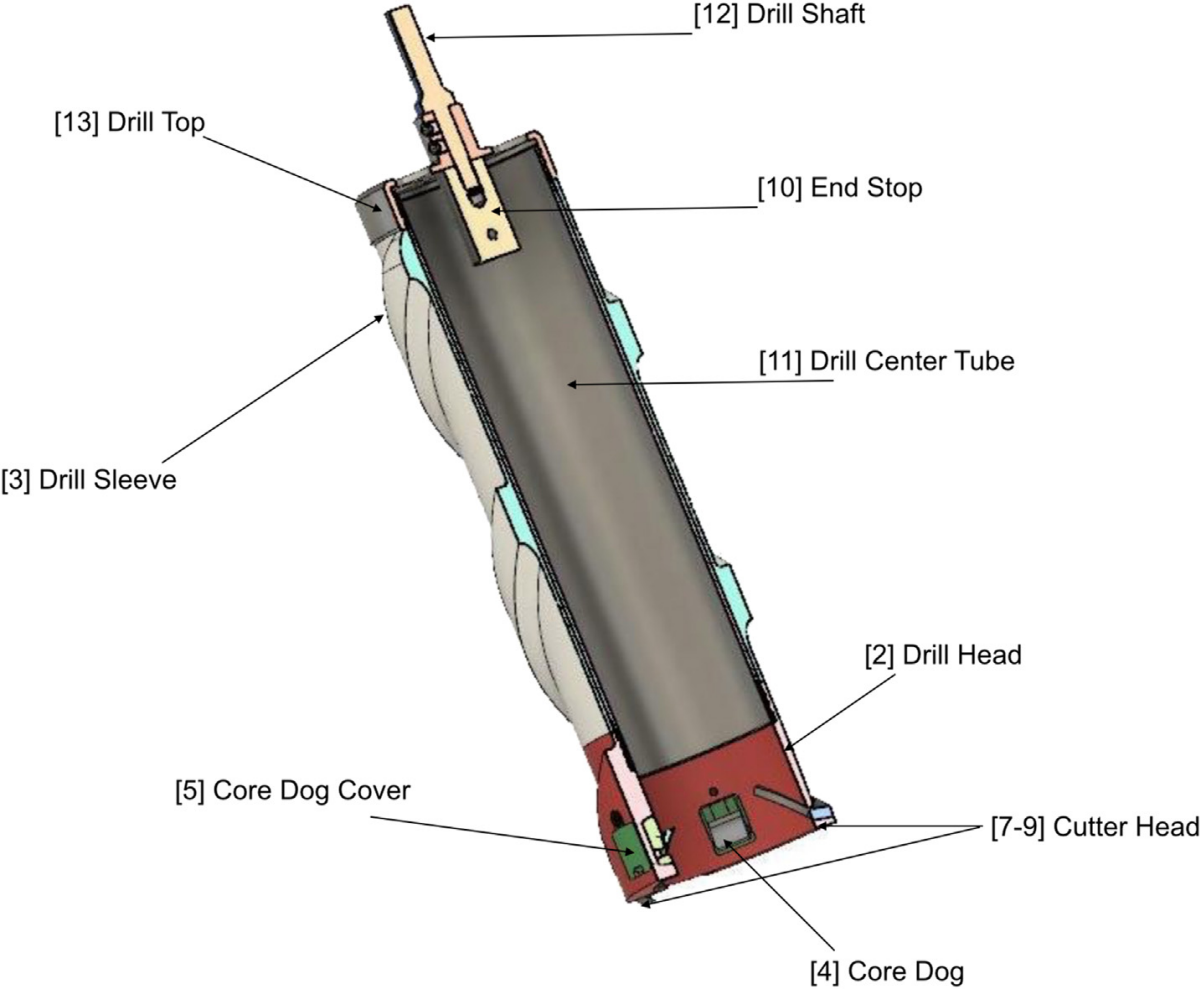
A cutaway view of the ice coring drill design with components labeled. Component names and numbering correspond to those found in the text.

Here we describe a lightweight and small ice coring drill that is simple to assemble and operate. Fig. 1 shows a rendering of the drill design from the top, bottom, and side. Fig. 2 shows a cross-section with the components labeled.

The pictures of the coring heads in [37] provided inspiration for the ice coring drill that is presented here. The final design was the result of iterative testing and optimization. While ice core cutting heads have been in use for decades [37], we were unable to obtain any for direct comparisons. As a result, we refrain from speculating on specific performance characteristics. We note that features like cutting teeth, core dogs, and spiral sleeves described in the remainder of this section are common to other ice coring drills in existence. Unlike previous designs, however, we have made all design files open source with the goal of promoting open scientific discovery.

The ice coring drill presented here was created directly from the design files using modern computer numerical control (CNC) machining and 3D printing techniques. We assume, perhaps incorrectly, that those engaged in the acquisition and analysis of ice cores have access to the resources needed to reproduce this ice coring drill, including machinists, CNC tools, 3D printers, and battery-operated hand drills, through their university, research institute, or place of employment.

With this assumption in place, the cost of the drill reflects the total cost of the raw materials and does not consider the costs of procuring, maintaining, and operating CNC machines and 3D printers, nor does it include the salary of those responsible for operating the aforementioned equipment as these costs are usually covered by institutional overhead and/or cost-sharing agreements between departments and/or institutions. Should this assumption prove to be incorrect, which is likely in some cases, interested parties can seek out the services of a private machine shop. If a private machine shop is unavailable or too costly, we invite those interested to contact the corresponding author. Furthermore, the authors make no attempt to account for regional differences in the prices of the raw materials. Finally, the cost estimate does not include the cost of a battery-operated hand drill as these drills are versatile pieces of equipment and are included in the toolkit of every expedition.

### 2.1 Cutting head

The cutting head (Fig. 1, Fig. 2) is 3D printed in nylon using selective laser sintering (SLS). SLS 3D printing is not required, however, and 3D printed parts can also be printed using more traditional 3D printers and filaments (e.g., PLA, PETG, or ABS). The cutting head holds three sets of machined steel cutting teeth, which are staggered to prevent icing [37], and are held in place using countersunk M3 head screws. The cutting head contains three spring-loaded ‘core dogs’ that hold the ice sample in place to prevent it from sliding out of the body of the core during retrieval.

### 2.2 Body

**Fig. 3:**
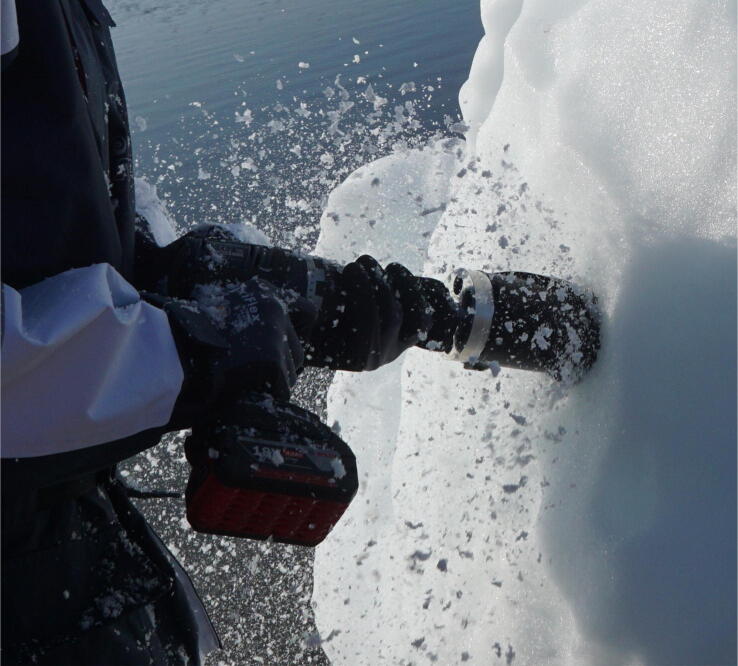
Sample acquisition underway on a grounded bergy bit in western Greenland. The sleeve efficiently removes ice cuttings. Note that the drill could be placed inside a plastic bag in warm weather if ice cuttings were to land and melt on the drill.

The main body of the drill is 191 mm long and is composed of inner and outer sleeves. The inner sleeve is a 70 mm diameter aluminium cylinder, which holds the ice sample. The outer sleeve is 3D printed in SLS nylon and uses spiral grooves [43] to remove ice cuttings (Fig. 3).

### 2.3 Drive Shaft

The drive shaft consists of a hexagonal bit that is machined from stainless steel and inserts into the chuck of the hand drill. The shaft assembly includes the drill top, which is machined from aluminium, and is threaded to hold the body of the drill. The end stop is machined from aluminum and prevents the ice core from reaching the top of the drill.

## 3 Design files

The design files include .STL and .STEP files for the 3D printed and machined parts, respectively. There are no metal alloy requirements for the machined parts. The 3D printed parts were constructed using SLS nylon, though they can also be constructed using PLA, PETG, and/or ABS, and the metal parts are machined using aluminium, stainless steel, spring steel, and knife steel, as indicated in the bill of materials table. For the most part, the file names are self explanatory and their placement is indicated in Fig. 2.

**Table:** 

Design filename	File type	Open source license	Location of the file
1. CoreDogSpring.STEP	CAD	CC BY 4.0	Mendeley Data
2. DrillHead.STL	CAD	CC BY 4.0	Mendeley Data
3. Drillsleeve.STL	CAD	CC BY 4.0	Mendeley Data
4. CoreDog.STEP	CAD	CC BY 4.0	Mendeley Data
5. CoreDogCover.STL	CAD	CC BY 4.0	Mendeley Data
6. Drillsleevepin.STEP	CAD	CC BY 4.0	Mendeley Data
7. Cutterheadinner.STEP	CAD	CC BY 4.0	Mendeley Data
8. Cutterheadmiddle.STEP	CAD	CC BY 4.0	Mendeley Data
9. Cutterheadouter.STEP	CAD	CC BY 4.0	Mendeley Data
10. Endstop.STEP	CAD	CC BY 4.0	Mendeley Data
11. Drillcentertube.STEP	CAD	CC BY 4.0	Mendeley Data
12. Drillshaft.STEP	CAD	CC BY 4.0	Mendeley Data
13. Drilltop.STEP	CAD	CC BY 4.0	Mendeley Data

## 4 Bill of materials

**Table:** 

Designator	Component	Number	Cost per unit currency	Total cost	Source of materials	Material type
1	Core dog spring	3	26.33	78.98	Machined	Metal (Spring Steel)
2	Drill head	1	26.33	26.33	3D Printed (SLS)	Polymer (Nylon PA12)
3	Drill sleeve	1	54.54	54.54	3D Printed (SLS)	Polymer (Nylon PA12)
4	Core dog	3	7.72	23.16	Machined	Metal (Knife Steel)
5	Core dog cover	3	9.53	28.58	3D Printed (SLS)	Polymer (Nylon PA12)
6	Drill sleeve pin	1	2.52	2.52	Machined	Metal (Stainless steel)
7	Cutter head inner	1	7.73	7.73	Machined	Metal (Knife Steel)
8	Cutter head middle	1	8.09	8.09	Machined	Metal (Knife Steel)
9	Cutter head outer	1	7.72	7.72	Machined	Metal (Knife Steel)
10	End stop	1	8.09	8.09	Machined	Metal (Aluminium)
11	Drill center tube	1	18.92	18.92	Machined	Metal (Aluminium)
12	Drill shaft	1	18.15	18.15	Machined	Metal (Stainless steel)
13	Drill top	1	20.57	20.57	Machined	Metal (Aluminium)
DIN 7991 M3 × 8–4.8 N	countersunk self-tapping screw	6	0.38	2.28	Accu.co.uk	Metal (Stainless steel)
DIN 912 M3 × 8–8 N	self-tapping screw	3	0.19	0.57	Accu.co.uk	Metal (Stainless steel)
DIN 912 M3 × 25–18 N	self-tapping screw	3	0.19	0.57	Accu.co.uk	Metal (Stainless steel)
DIN 916 M5 × 6-N	set screw	4	0.15	0.60	Accu.co.uk	Metal (Stainless steel)

### 4.1 Build instructions

Before commencing assembly of the drill ensure that the following criteria are met for the manufactured parts. Ensure that the M74 × 1.0 threading on the drill top (13; Fig. 2), drill head (2; Fig. 2), and drill sleeve (3; Fig. 2) are machined together for optimal fits. Ensure that the cutting teeth and core dogs have been hardened, tempered, and sharpened according to the best practices for the selected knife steel. Sharp cutting edges ensure optimal operation and longevity of the drill. The outer surfaces of the drill head and outer sleeve, and inner surface of the inner sleeve, should be coated in a layer of ski wax after assembly to prevent ice from sticking to the surface. Ensure that all sharp edges on the non-cutting parts are de-burred to prevent injury during assembly and operation.

### 4.2 Build tips

Always refer back to the assembly files provided for the orientation and number of parts. Use caution when handling the core dogs and cutting teeth and always wear protective gloves when handling sharp items.

### 4.3 Cutting head assembly

Use a sacrificial M3 self-tapping screw to tap out the screw holes for the cutting teeth (7–9; Fig. 2), core dog swivel, and core dog spring retaining screws. Next, install the core dogs (4; Fig. 2) in their covers by sliding the M3 × 25–18 N swivel screw through the core dog and threading it into the drill head; ensure that the cutting edge is facing inwards. DO NOT OVER-TIGHTEN! Insert the core dog springs on top of the core dogs and screw down the retaining M3 × 8–8 N screws snugly; ensure that the screws are not protruding into the center cavity of the drill. Next, slide the core dog covers (5; Fig. 2)) into the core dog pockets. The covers should snap firmly into place around the swivel screws and sit flush with the outer face of the drill head. Ensure that the core dogs swivel freely and have a firm spring action when pressed from the inside. BE CAREFUL AROUND CUTTING EDGES! Insert the drill sleeve pins into their pockets in the upper face of the drill head. These should set firmly into place. If not, secure them with a drop of cyanoacrylate super-glue. Do not install cutting teeth yet and set the drill head aside for now.

### 4.4 Body assembly

Ensure that the drill center tube (11; Fig. 2) fits into the drill sleeve with a snug sliding fit. If the fit is too tight then sand the inside of sleeve (3; Fig. 2) lightly until a satisfactory fit is achieved. Slide the drill sleeve (3; Fig. 2)) onto the drill center tube (11; Fig. 2), leaving the two flush on the end of the drill sleeve with its three sleeve pin pockets, and set the assembly aside.

### 4.5 Drive shaft assembly

Ensure that the drill shaft (12; Fig. 2) inserts into the drill top (13; Fig. 2) snugly without forcing the shaft into it, leaving no gap between drill shaft and drill top. Next, secure the drill shaft (12; Fig. 2)) to the drill top (13; Fig. 2) with four M5 × 6-N set screws inserted into the corresponding four threaded holes and tighten down the set screws firmly. Thread the end stop (10; Fig. 2)) onto the exposed end of the shaft on the underside of the drill top (13; Fig. 2). Tighten down firmly by using a 4 mm Allen key through the diagonal hole as leverage. Set the drill shaft assembly aside for the final assembly of the drill.

### 4.6 Final assembly

Begin by placing the cutting head assembly on a level surface, with the threaded end facing up. Next, fit the body assembly onto the cutting head assembly by fitting the drill sleeve onto the protruding drill sleeve pins. Gently press down on the drill center tube and rotate it with one hand while holding the drill head with the other to engage the threading. The two should thread together with minimal force. Avoid cross-threading, and repeat this step if in doubt. Ensure that the drill sleeve fits squarely against the drill head with no gaps. Thread the drill center tube into the cutting head assembly until fully seated. With the new assembly stood on a level surface, thread the drive shaft assembly onto the exposed threaded section of the drill center tube. The two should engage smoothly with minimal force. If not, lubricate the threads with a small amount of silicone grease. Thread the drive shaft assembly onto the assembly until fully seated, gripping the drill head with one hand and the drill top with the other. Ensure that there are no gaps between the drill top and drill sleeve and the sleeve is held snugly in place. If not, ensure that the center tube is fully seated in either the drill head or drill top. Lay the drill assembly down on a flat surface, supported enough as to not roll away. Install the cutting teeth by placing them onto the mounting surfaces. Secure each cutting tooth with two M3 × 8–4.8 N countersunk screws. Ensure that the teeth are flush with the inner surface of the drill head. Tighten down screws snugly, but do not over-tighten! Cover the cutting teeth by draping a piece of cloth over the cutting end of the drill and securing with an elastic band.

## 5 Operation instructions

### 5.1 Warnings

• WARNING – icebergs can be unstable and unpredictable. Approach with extreme caution and only under the supervision of experienced personnel.
• WARNING – Never hold the drill by its flutes or head when mounted in an electric hand drill; serious injury may follow.
• CAUTION – always wear proper protective equipment; protective gloves and safety glasses as a minimum when drilling.
• CAUTION – cutting teeth are sharp and should be covered when not in use to avoid injury and/or damage.
• CAUTION – Always have a trained second person standing by and attentive when operating the drill.
• ATTENTION – Faster drilling speeds have been shown to produce better results. Ensure that the intended electric hand drill can reach minimum 1500 RPM.

### 5.2 Assembly before use

Drape a plastic bag over the electric hand drill to protect from water damage from ice cuttings. If the ice coring drill is already assembled, proceed to the next section. Align the threads on the ice coring drill top with threads on the body of the ice coring drill. Slowly screw the two parts together and make sure to avoid cross-threading. Screw down snugly. Insert the hexagonal shaft into the bit holder on the electric hand drill. Tighten the hand drill’s chuck to secure the hexagonal shaft. Verify correct rotation - keep clear of people and other equipment. The drill should now be handled with extreme care and treated as dangerous.

### 5.3 Drilling

**Fig. 4:**
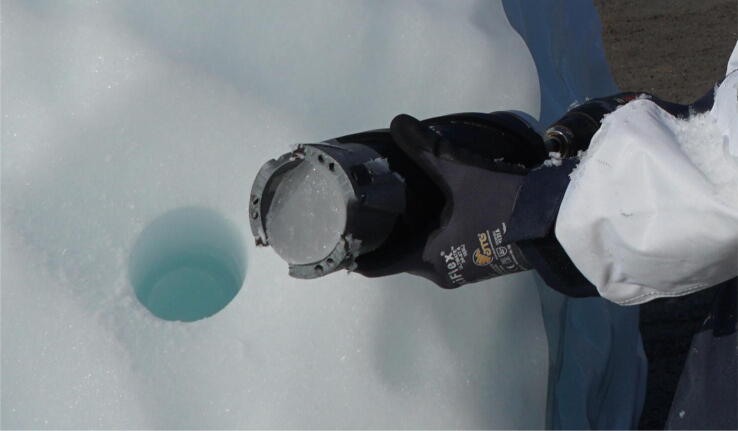
A detached core held in place by the core dogs with a clean borehole visible in the background.

Place the ice coring drill squarely against the iceberg with all 3 sets of cutting teeth engaged. Begin slowly rotating to form a groove to guide the cutting teeth. After a 5 mm groove has been formed, slowly increase to full speed – faster is better – slow drilling can get stuck (Fig. 3). Let the drill do the work and only apply enough pressure to keep the assembly stable and advancing into the ice. If the ice coring drill stops advancing before reaching its end stop, remove it and check for ice build-up and inspect cutting teeth. If ice is found; move a safe distance from iceberg, remove the ice coring drill from the electric hand drill and clean the cutting teeth. After the drill reaches maximum penetration and the sample reaches the end stop, wiggle the drill until you feel a snap. The ice coring drill should now be free to move inside the hole. Remove the ice coring drill with the sample inside (Fig. 4). Move the boat a safe distance away from the iceberg.

### 5.4 Sample collection

**Fig. 5:**
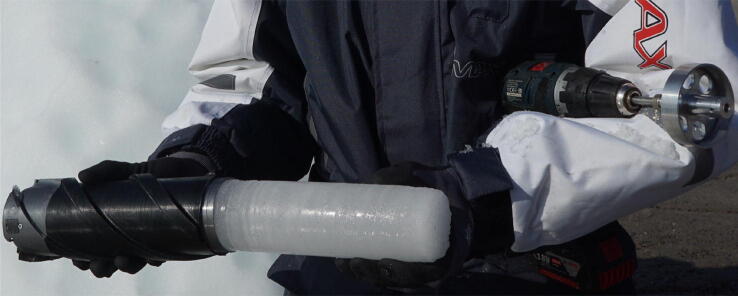
After drilling and removing the core, the shaft is detached from the drill and the sample is extracted. Here, the ice sample is displayed, but normally it would be placed directly into a sterile plastic bag and into a freezer for later analysis.

Note that anyone handling the ice sample should wear sterile gloves to prevent contamination if sampling for microbes. Loosen the top of the ice coring drill with a quarter twist. With the electric hand drill set to its slowest speed, slowly unscrew the body of the ice coring drill from the drill top. Make sure to point the cutting teeth away from any people or equipment (Fig. 5). Once unscrewed, set the hand drill and drill top aside. Position a labeled and sterile plastic bag over the now exposed end of the core. Use a sturdy tool to push the core into the bag. Be sure to keep hands away from cutting teeth. Seal the bag and store the sample in a freezer for later processing.

## 6 Validation and characterization

### 6.1 Field trials

The ice coring drill system was tested during the ‘Vaigat Iceberg – Microbial Oil degradation and Archaeological heritage investigation’ (VIMOA). VIMOA took place along the Nuussuaq peninsula (north of Disko Island) from 29 July to 16 August, 2019. VIMOA was a multidisciplinary research cruise that investigated iceberg drift and deterioration, microbial assemblages and microbial oil degradation, and coastal archaeological sites situated near natural oil seeps [44]. The ice coring drill was used to extract 11 samples in total from three freely drifting icebergs and three bergy bits that were stranded on shore. The ice sampling procedure is demonstrated in Fig. 3, Fig. 4, Fig. 5. The sampling included a variety of ice types, including white, blue, sediment-rich, and ice with a firn layer. Ice sample collection took <30 s for each sample. The samples will be analyzed for microbial assemblages, nutrients (nitrate, phosphate, ammonia, and silica), and iron. The ice samples will also be used to compute the ice density, which will be used in the estimation of iceberg deterioration rates.

### 6.2 Future improvements

While the ice coring drill performed well during sampling, sample extraction was awkward at times. We suggest the following improvements for future versions. First, a device that immobilizes the body of the drill and, therefore the cutting teeth, which do not discriminate between ice and human flesh, while it is unscrewed from the top. Second, a plunger of some sort would be helpful for extracting the ice sample. Finally, a storage sheath should be constructed to protect the cutting teeth during transport. The envisioned improvements could be designed to cover the cutting teeth while immobilizing the drill and still allowing for sample extraction, thereby also making sample extraction safer and easier.

## Declaration of Competing Interest

The authors declare that they have no known competing financial interests or personal relationships that could have appeared to influence the work reported in this paper.
